# Research Hotspots and Emerging Trends of Orthodontic‐Related Discomfort and Pain: A Bibliometric Review

**DOI:** 10.1155/prm/3757286

**Published:** 2025-12-05

**Authors:** Shirui Bai, Sining He, Zhenrong Yin, Yiliu Zhou, Fei Yu, Zhihe Zhao, Peilin Li

**Affiliations:** ^1^ State Key Laboratory of Oral Diseases, National Center for Stomatology, National Clinical Research Center for Oral Diseases, West China School of Stomatology, Sichuan University, Chengdu, China, scu.edu.cn; ^2^ Department of Orthodontics, West China Hospital of Stomatology, Sichuan University, Chengdu, China, scu.edu.cn

## Abstract

**Background:**

Given the increasing volume of scientific research on orthodontic‐related discomfort and pain (ORDP) in recent years and the scarcity of related quantitative analyses, this study aims to analyze the research hotspots and emerging trends of ORDP with the bibliometric method.

**Materials and Methods:**

A systematic search within the Web of Science Core Collection database was conducted on 30th April 2024. After manually screening and removing duplicate or irrelevant publications, we collected relevant studies for comprehensive bibliometric analysis, encompassing analyses of countries, institutions, authors, journals, documents, and keywords, which were performed using VOSviewer 1.6.19 and CiteSpace 5.7R5.

**Results:**

A total of 970 publications were included in the bibliometric analysis. A significant upward trend in the annual publication output was observed, peaking in 2020. China (*n* = 120) has the highest publication output, representing 12.9% of the documents, while the United States has the highest citation count (2778). Sichuan University (*n* = 47), contributing 5.08% of all publications, accounts for the largest volume of literature in the ORDP research field. The American Journal of Orthodontics and Dentofacial Orthopedics (*n* = 91) was identified as the most productive journal in this domain. Furthermore, a comprehensive keyword analysis revealed five clusters for ORDP research and provided insights into the hotspots and trends. ORDP associated with invisible orthodontics, laser therapy to manage ORDP, and ORDP related to accelerated orthodontic tooth movement surgery are burgeoning areas of research interest.

**Conclusion:**

This study reveals the global research distribution in the field of ORDP over time and across regions, highlighting key contributors across various dimensions. The field of ORDP has experienced significant growth, with emerging interests in invisible orthodontics, laser therapy for ORDP management, and accelerated orthodontic techniques. Further high‐quality research is needed to explore personalized appliance selection, optimize innovative orthodontic technique processes, and establish specific protocols for promising management strategies such as low‐level laser therapy.

## 1. Introduction

Orthodontic treatment, mainly aimed at aligning teeth, correcting malocclusion, improving facial aesthetics, and improving airway patency, contributes to higher oral health–related quality of life (OHRQoL) [1–3]. Orthodontic‐related discomfort and pain (ORDP) is a common complaint among patients undergoing orthodontic treatment, with significant effects on patient experience and even treatment continuation [4]. Approximately 65.7% of patients report general dentogingival discomfort and 34.3% report localized pain during the early stages of fixed appliance (FA) therapy, with intensity often described as moderate to severe [5, 6]. These symptoms, typically presenting as dull pain around the teeth or a generalized sense of unease, usually begin within 2 hours of appliance or archwire placement and persist for 5–7 days [7]. As such, ORDP is among the main factors affecting patient satisfaction and adherence to treatment protocols [8].

The biological mechanisms underlying ORDP involve an interplay of cellular, vascular, and molecular events initiated by the application of orthodontic forces. These events lead to inflammatory responses, with pain signals ultimately modulated in the brain to elicit emotional and cognitive responses [9]. Pharmacological interventions, such as nonsteroidal anti‐inflammatory drugs (NSAIDs), have demonstrated efficacy in mitigating postoperative orthodontic pain [10]; however, they carry risks including potential gastrointestinal toxicity and allergic reactions [11]. This has led to a growing interest in nonpharmacological strategies to alleviate ORDP, particularly the emergence of laser therapy through photobiomodulation as a novel approach in recent years [12, 13]. Concurrently, the advent and application of invisible orthodontics and accelerated orthodontic technologies have brought new opportunities and changes to orthodontic treatment.

Bibliometric analysis provides a systematic approach to meet the need for a comprehensive overview by quantifying and visually mapping the intellectual structure of the field and tracing its thematic evolution. Previous bibliometric studies have mapped research trends in early orthodontic treatment [14] and analyzed publication patterns in orthodontic journals [15]. Regarding orofacial pain, bibliometric analyses have also been conducted in relation to temporomandibular disorder research [16] and other orofacial pain conditions. However, to our knowledge, no bibliometric analysis has yet been conducted on ORDP, indicating a clear gap that this study aims to fill. Accordingly, the present study aims to (1) identify influential contributors (countries, institutions, authors, journals, and documents), (2) map research themes and temporal trends, and (3) highlight emerging topics with potential to guide future research directions.

## 2. Materials and Methods

### 2.1. Database Search and Literature Screening

A literature search was conducted in the Web of Science Core Collection. After research and group discussion, the search query was set as “((TS = (discomfort) OR TS = (pain)) AND TS = (orthodontic)).” The TS field encompasses the title, abstract, author keywords, and Keywords Plus, as per the Web of Science indexing criteria. This search was conducted without constraints on publication type or publication year, ensuring a comprehensive and inclusive retrieval of relevant literature. The search was conducted, and the data were exported for subsequent analysis on 30th April 2024.

Duplicated publications were excluded. Two researchers (Shirui Bai and Sining He) independently evaluated the title, abstract, and full text (if necessary) to filter out irrelevant publications. Publications were included if they addressed ORDP as either the primary research question or a major reported outcome. Relevant clinical and laboratory studies investigating the mechanisms, management, or patient‐reported outcomes of ORDP were considered. Only articles published in English were eligible. No restrictions were applied regarding publication year or document type. Only records with complete bibliographic information (title, abstract, author, keywords, and source) were retained. Publications were excluded if they were unrelated to orthodontics, did not examine discomfort or pain in the orthodontic context, or represented duplicate records. Any discrepancies in the evaluation were addressed through consensus discussions involving a third researcher (Peilin Li). Ultimately, relevant papers were identified for comprehensive bibliometric analysis.

### 2.2. Bibliometric Analysis and Visualization

Descriptive statistics (e.g., citations per document [CPD], citations per year [CPY]) were computed using Microsoft Excel 2016. Total link strength (TLS) values were generated in VOSviewer, where TLS is defined as “the total strength of an item’s links with other items” in the map, with higher values indicating stronger or more frequent collaborative connections [17]. Data visualization was performed via GraphPad Prism 8.4.3. Bibliometric analysis was conducted via VOSviewer 1.6.19 and CiteSpace 5.7R5. VOSviewer was utilized for analyzing and visualizing data across countries, institutions, authors, and keywords. CiteSpace was employed for country collaboration network, clustering, and bursting analyses. SCImago Graphica 1.0.43 was used to visualize the geographic distribution of publications.

For coauthorship and institutional analyses, only authors with more than three publications and institutions with at least seven publications were included to ensure the representation of significant contributors. Given the size of the dataset (*n* = 970) and the technical complexity of systematically identifying self‐citations, we retained them in our analysis. However, this factor should be considered when interpreting the results.

Keyword analysis was refined by consolidating synonymous terms, such as singular and plural forms, nouns and verbs, abbreviations and full terms, as well as trade names and generic names, into a single representative term. For instance, the abbreviation “TMD” was unified under the term “temporomandibular joint disorders.” The comprehensive consolidated datasheet is provided in the supporting information (Table S1). In addition, only keywords with more than 10 occurrences were included.

## 3. Results

### 3.1. Annual Publication Amount

After our screening and filtering from the original 1576 literature, the descriptive statistics encompassed a total of 970 publications, predominantly articles (*n* = 786) and reviews (*n* = 140) (Figure 1(a)). The trend of the annual publication amount was characterized by an initial phase with fewer than 10 publications per year prior to 2007, followed by a significant increase, peaking at 112 research papers in 2020 (Figure 1(b)).

Figure 1(a) Types of publications. (b) Annual and cumulative publications of research about ORDP. The red line represents the annual publications, while the blue line shows the cumulative.

**Figure:**
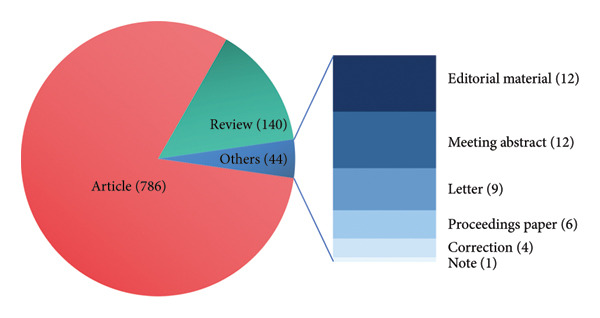
(a)

**Figure:**
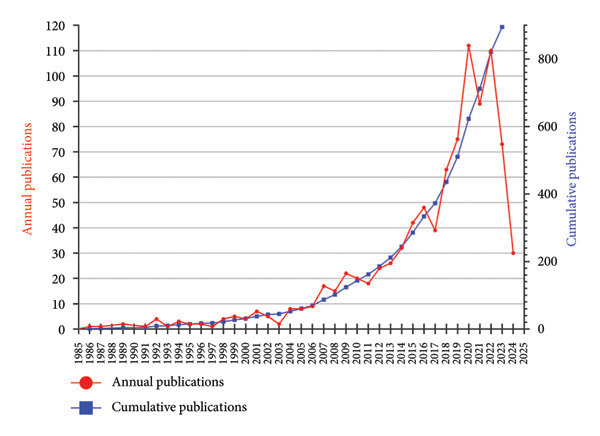
(b)

### 3.2. Countries

A collective of 73 countries had contributed to the field of ORDP, with 27 countries having more than 10 publications each. The top 10 most productive countries are listed in Table 1. China had the highest publication output (*n* = 120), constituting 12.9% of the total. The United States ranked second in production (*n* = 100) but led in citation count with 2778 citations. In addition, the CPD is employed to evaluate the research impact, and the TLS demonstrates the collaboration condition among these countries in the field of ORDP.

**Table 1:** Top 10 most productive countries.

Rank	Country	Documents	Percentage (%)	Citations	CPD	TLS
1	China	120	12.9	1729	14.4	38
2	USA	100	10.8	2778	27.7	64
3	Brazil	84	9.0	908	10.8	21
4	India	78	8.4	425	5.4	14
5	Italy	67	7.3	905	13.5	28
6	England	62	7.2	1397	22.5	60
7	Japan	58	6.1	1250	21.5	11
8	Türkiye	54	6.2	1027	19.0	4
9	Saudi Arabia	52	5.6	486	9.3	46
10	Iran	39	4.2	359	9.2	16

Figure 2(a) visually represents the global distribution of relevant publications with a world map, and Figure 2(b) presents a density map of international collaboration. In Figure 2(c), the temporal distribution of publications is denoted by “tree rings,” and the color gradient of these rings corresponds to the publication years, providing a visual timeline of research activity. The centrality of the United States (0.42), England (0.35), Saudi Arabia (0.21), China (0.15), and Brazil (0.12) in the global research network was highlighted by the purple markers.

Figure 2Collaboration analysis of countries. (a) World map of the publications across countries/regions. The sizes of the dots indicate the number of publications, and the colors represent the citation counts. (b) Density map of the collaboration groups among countries, with the color depth representing the publication counts of the countries. (c) Collaboration network of the countries. The “tree ring” shows the publications over the time scale. The ring thickness represents the number of publications.

**Figure:**
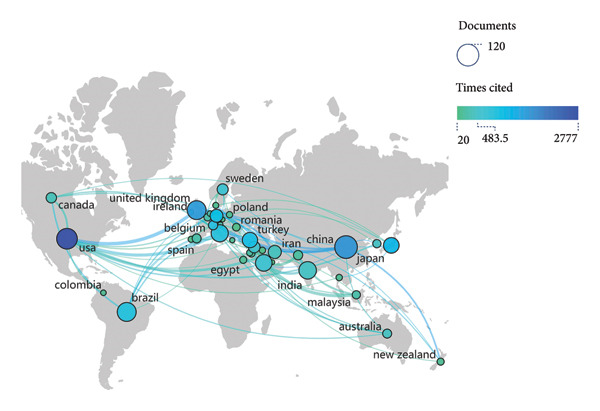
(a)

**Figure:**
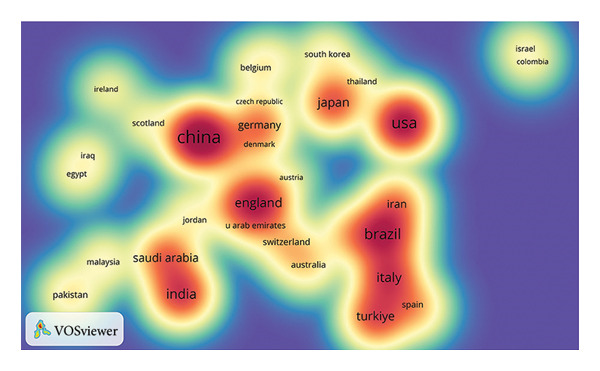
(b)

**Figure:**
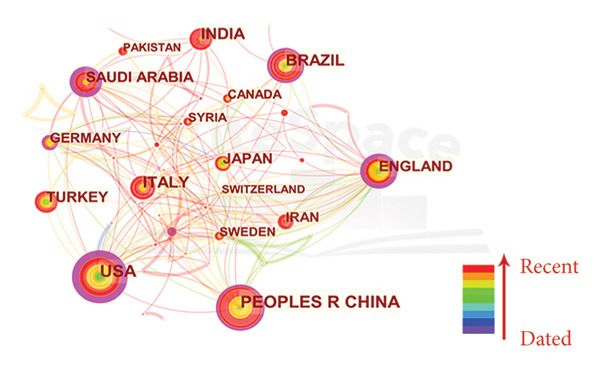
(c)

### 3.3. Institutions

Table 2 presents the top 10 most productive institutions globally. A total of 61 institutions contributed more than five publications to the field of ORDP, with Sichuan University ranking first with 47 publications. The University of Sao Paulo and the University of Damascus followed with 27 and 25 publications, respectively. Despite having relatively lower publication volumes, the University of Bern, The Ohio State University, and Okayama University garnered considerable citations. Nihon University, despite having a lower volume of publications, achieved the highest CPD of 33.1. In terms of collaboration, Damascus University had the highest TLS (TLS = 10). The collaborative network (Figure 3(a)) and density map (Figure S1) further illustrated the research cooperation among institutions. Several research clusters were detected, but with limited interconnections, suggesting a preference for forming targeted partnerships over broad global collaborations. While such a strategy may enable more intensive collaboration and facilitate deep exploration of specific topics, the lack of wider global linkages could restrict the diversity and scope of research directions.

**Table 2:** Top 10 most productive institutions.

Rank	Organization	Country	Documents	Percentage	Citations	CPD	TLS
1	Sichuan Univ	China	47	5.0%	791	16.8	9
2	Univ Sao Paulo	Brazil	27	2.9%	291	10.8	1
3	Univ Damascus	Syria	25	2.7%	209	9.9	10
4	Nihon Univ	Japan	14	1.5%	463	33.1	1
5	Univ Naples Federico II	Italy	12	1.3%	281	23.4	7
6	Univ Manchester	United Kingdom	12	1.3%	250	20.8	5
7	Univ Toronto	Canada	11	1.2%	136	12.4	4
8	Univ Hong Kong	China	10	1.1%	282	28.2	0
9	King’s College London	United Kingdom	10	1.1%	217	21.7	9
10	Univ Otago	New Zealand	10	1.1%	98	9.8	4

Figure 3(a) Collaborative network of institutions. The nodes represent the institutions, with larger sizes indicating more publications, and the lines show the collaboration. (b) Coauthorship mapping. The nodes represent the authors, with larger sizes reflecting more publications, and the lines show the collaboration.

**Figure:**
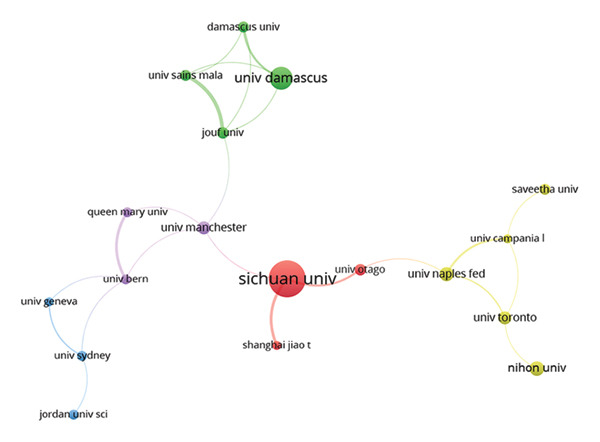
(a)

**Figure:**
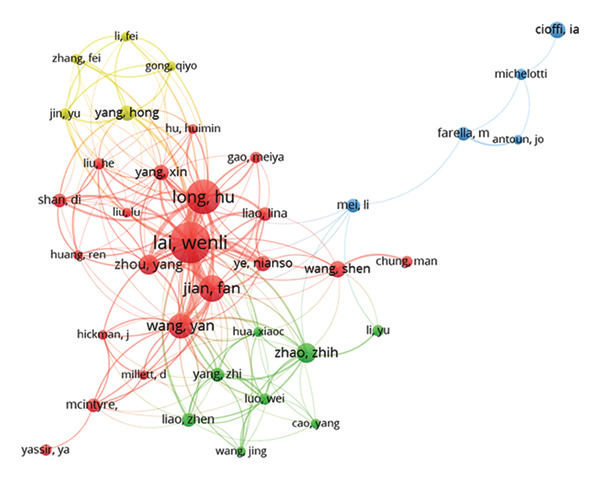
(b)

### 3.4. Authors

A total of 179 authors contributed to three or more publications in this field. Table 3 lists the 10 most prolific authors in the realm of ORDP research. Lai Wenli was the most productive author, with a total of 28 publications, followed by Hajeer Mohammad (*n* = 23) and Long Hu (*n* = 21). Lai Wenli also had the highest number of citations (411) and the top TLS (99). Figure 3(b) illustrates the coauthorship network. Few multinational author groups were detected; most author groups consisted of authors from the same institution and country. For example, 6 of the 10 most prolific authors were from the same institution, Sichuan University (Table 3).

**Table 3:** Top 10 most productive authors.

Rank	Author	Institution, country	Documents	Citations	CPD	*H*‐index^∗^	TLS
1	Lai, Wenli	Sichuan University, China	28	411	14.7	26	99
2	Hajeer, Mohammad	Univ Damascus, Syria	23	230	10.0	19	21
3	Long, Hu	Sichuan University, China	21	357	17.0	24	76
4	Jian, Fan	Sichuan University, China	14	314	22.4	19	60
5	Wang, Yan	Sichuan University, China	13	304	23.4	13	56
6	Alam, Mohammad Khursheed	Jouf University, Saudi Arabia	13	151	11.6	24	12
7	Burhan, Ahmad S.	Univ Damascus, Syria	11	39	3.5	10	16
8	Zhou, Yang	Sichuan University, China	9	188	20.9	13	46
9	Zhao, Zhihe	Sichuan University, China	9	179	19.9	37	20
10	McGrath, Colman	Univ Hong Kong, China	8	238	29.8	45	18

^∗^Data were derived from the Web of Science author profile.

### 3.5. Journals

Table 4 presents the top 10 most productive journals in the field of ORDP. The American Journal of Orthodontics and Dentofacial Orthopedics led with 91 publications, closely followed by Angle Orthodontics with 84 publications and the European Journal of Orthodontics with 62 publications. Collectively, these three journals contributed 237 articles, a sum that doubles the total of the next seven journals combined.

**Table 4:** Top 10 journals with the most documents published.

Rank	Source	Documents	Citation	CPD	JCR^∗^	IF (2023)^∗^	IF (5 years)^∗^
1	American Journal of Orthodontics and Dentofacial Orthopedics	91	4173	45.8	Q1	2.7	3.2
2	Angle Orthodontist	84	2606	31.0	Q1	3.0	3.3
3	European Journal of Orthodontics	62	1904	30.7	Q1	2.8	3.0
4	Cureus Journal of Medical Science	27	65	2.4	Q3	1.0	1.1
5	BMC Oral Health	21	163	7.8	Q1	2.6	3.2
6	Lasers in Medical Science	19	627	33.0	Q2	2.1	2.4
7	Orthodontics and Craniofacial Research	18	165	9.2	Q2	2.4	2.7
8	Journal of Orofacial Orthopedics	16	137	8.6	Q3	1.3	2.0
9	Progress in Orthodontics	15	234	15.6	Q1	3.5	4.5
10	Journal of Oral Rehabilitation	15	232	15.5	Q1	3.1	3.9

^∗^The data were derived from the Web of Science journal page.

### 3.6. Papers

Table 5 shows the top 10 most cited papers. The article “Clinical use of miniscrew implants as orthodontic anchorage,” [18] authored by Kuroda et al. in 2007, ranked first and occupied a prominent position in the ORDP literature, underscoring its foundational contribution to the field. In addition, the article “Effect of micro‐osteoperforations on the rate of tooth movement [19]” had the highest CPY of 22.0, indicating its sustained influence. Overall, the top 10 papers were mainly from two influential journals, the American Journal of Orthodontics and Dentofacial Orthopedics and the European Journal of Orthodontics.

**Table 5:** Top 10 most cited papers.

Rank	Author	Article title	Source	Document type	Times cited	CPY
1	Kuroda (2007)	Clinical use of miniscrew implants as orthodontic anchorage: success rates and postoperative discomfort	American Journal of Orthodontics and Dentofacial Orthopedics	Article	338	19.9
2	Ngan (1989)	Perception of discomfort by patients undergoing orthodontic treatment	American Journal of Orthodontics and Dentofacial Orthopedics	Article	313	8.9
3	Scheurer, P. A. (1996)	Perception of pain as a result of orthodontic treatment with fixed appliances	European Journal of Orthodontics	Article	299	10.6
4	Krishnan, V. (2007)	Orthodontic pain: from causes to management–a review	European Journal of Orthodontics	Review	277	19.8
5	Erdin, A. M. E. (2004)	Perception of pain during orthodontic treatment with fixed appliances	European Journal of Orthodontics	Article	259	13.0
6	Motoyoshi, M. (2007)	Effect of cortical bone thickness and implant placement torque on stability of orthodontic mini‐implants	International Journal of Oral and Maxillofacial Implants	Article	244	14.4
7	Jones, M. (1992)	The pain and discomfort experienced during orthodontic treatment–a randomized controlled clinical trial of 2 initial aligning arch wires	American Journal of Orthodontics and Dentofacial Orthopedics	Article	227	7.1
8	Alikhani, M. (2013)	Effect of micro‐osteoperforations on the rate of tooth movement	American Journal of Orthodontics and Dentofacial Orthopedics	Article	242	22
9	Sergl, H. G. (1998)	Pain and discomfort during orthodontic treatment: causative factors and effects on compliance	American Journal of Orthodontics and Dentofacial Orthopedics	Article	216	8.3
10	Youssef, M . (2008)	The effect of low‐level laser therapy during orthodontic movement: a preliminary study	Lasers in Medical Science	Article	196	12.3

### 3.7. Keywords

Our analysis yielded a total of 2635 keywords. The top 10 most common keywords were pain (334), discomfort (267), perception (225), orthodontic tooth movement (213), orthodontic treatment (171), orthodontics (169), efficacy (83), management (81), appliance (74), and therapy (73).

**Figure 4:**
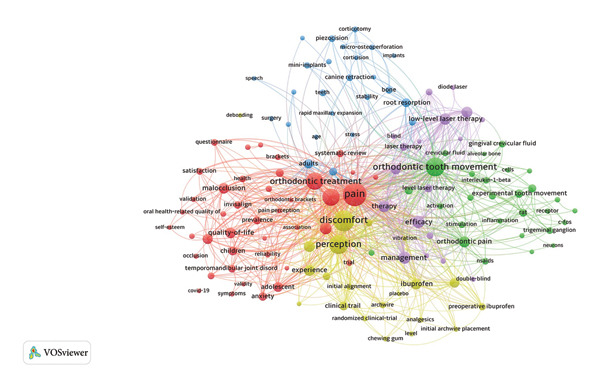
Cluster analysis of keyword co‐occurrence networks. The nodes represent keywords, and the size corresponds to the frequency of occurrence. Concurrent keywords are connected by lines whose thickness shows the intensity of co‐occurrence. Keywords that are clustered together share the same color.

Figure 4 illustrates the co‐occurrence clustering network of keywords, with a threshold set for terms occurring more than 10 times. The node size corresponds to the frequency of term occurrence within the database, and clusters are distinguished by color to represent different research themes. The detailed keyword composition of each cluster is provided in additional information (Table S2).

The five clusters are as follows:

1. The red cluster highlights appliance types and evaluation perspectives of ORDP, including “brackets,” “Invisalign,” “quality of life,” and “anxiety.”
2. The green cluster focuses on the study of biological events behind ORDP, with keywords such as “experimental tooth movement,” “inflammation,” and “trigeminal ganglion.”
3. The blue cluster includes a variety of specific orthodontic techniques, such as “mini‐implants,” “corticotomy,” “piezocision,” and “rapid maxillary expansion.”
4. The yellow cluster aggregates terms related to medications for pain management, such as “ibuprofen,” “acetaminophen,” “placebo,” and “clinical trials.”
5. The purple cluster centers on nonpharmaceutical approaches for pain relief, featuring terms such as “low‐level laser therapy,” “irradiation,” and “photobiomodulation therapy.”

Figure 5 was generated to indicate the temporal trends and burst dynamics of keywords regarding the research field. Time‐zone visualization (Figure 5(a)) delineates the temporal trajectory of the keywords, highlighting both foundational concepts (e.g., discomfort and malocclusion) and emerging themes (e.g., low‐level laser and piezosurgery). Figure 5(b) displays the top 25 keywords with the strongest citation bursts, identifying those that experienced a statistically significant surge in usage over a short period. Together, Figures 5(a) and 5(b) offer complementary perspectives that enhance the understanding of the evolution and focus shift of research in this field.

Figure 5Temporal trends and burst dynamics of keywords. (a) Time‐zone map of the keywords. Keywords are placed according to the time of occurrence, with recent keywords placed on the upper right of the figure. The lines reflect the co‐occurrence, with colors representing the average time of co‐occurrence. (b) Top 25 keywords with the strongest citation bursts. The red lines illustrate the duration of the citation burst.

**Figure:**
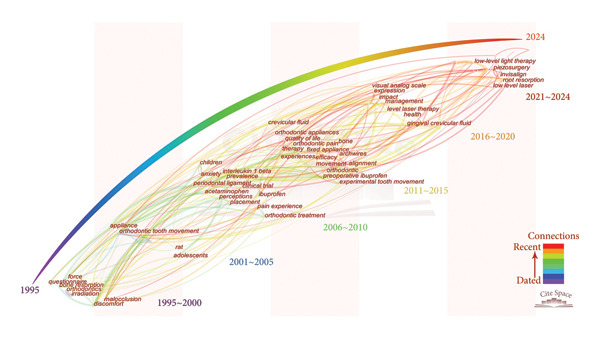
(a)

**Figure:**
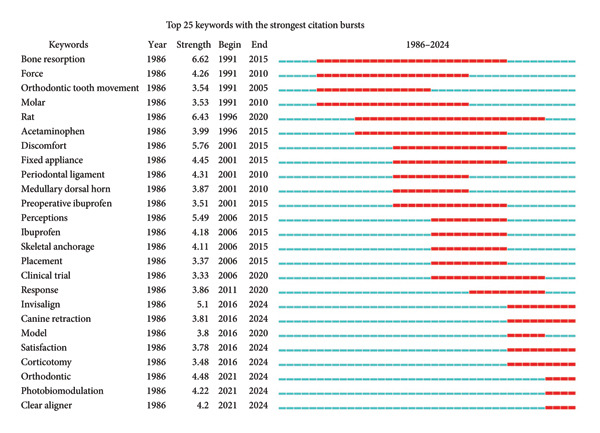
(b)

In summary, the results indicate that the hotspots of ORDP‐related research have evolved significantly. Initially focused on the characteristics of ORDP and fundamental physiological aspects of orthodontic treatment, there is now a discernible shift toward innovative techniques. This trend is marked by increasing interest in advanced methods such as piezosurgery, clear aligners (CAs), and laser therapy.

## 4. Discussion

Previous bibliometric studies on pain and discomfort in the orofacial region have primarily examined conditions such as temporomandibular disorders, bruxism, and myofascial pain [20–22]. While these conditions also involve pain and discomfort, ORDP mainly arises from treatment‐induced mechanical forces and therefore represents a distinct research focus. To the best of our knowledge, this is the first bibliometric analysis dedicated specifically to ORDP, thereby providing a focused perspective on its scholarly development and highlighting the growing global attention to this topic.

Most orthodontic treatment inherently relies on the placement of various appliances to achieve controlled tooth movement. Although effective in correcting malocclusion, these appliances are often associated with varying degrees of discomfort and pain. Driven by the need to improve patient compliance and prioritize patient‐centered outcomes, the clinical imperative to alleviate ORDP has stimulated research into its characteristics, mechanisms, and management strategies. This growing interest is reflected in the marked increase in publications in recent years (Figure 1(b)).

Our findings reveal the global distribution of research activity in the field of ORDP. First, we underscored the significant contribution of China in terms of publication volume while highlighting the leadership of the United States in citation impact. The discrepancy between the most prolific country (China) and the most cited country (USA) may be explained by temporal publication patterns. China’s rapid increase in research output is a relatively recent phenomenon, as suggested by the prominent red and orange ring around its node in Figure 2(c), indicating more recent publications with limited time to accumulate citations. In contrast, the USA has maintained a leading role for a longer period, allowing its publications to accrue citations over time and thereby achieving a higher overall impact. Among institutions, Sichuan University stands out, contributing 5.08% of all publications. Its prominence is further emphasized by the most prolific author, Lai Wenli, and 5 other top 10 productive authors being affiliated with this university. Despite having lower publication numbers, the University of Bern, The Ohio State University, and Okayama University have garnered considerable citations. These citation patterns may reflect multiple factors that contribute to the research impact, including the significance of the research questions addressed, the methodologies employed, and the extent to which the findings resonate with the broader academic community.

Among the most cited works, the article by Kuroda et al. [18] on the clinical use of miniscrew implants for orthodontic anchorage has been pivotal, identified as the most cited paper within our dataset. The article explored the effectiveness of miniscrews while highlighting the accompanying pain and discomfort with mucoperiosteal incision or flap surgery before miniscrew placement. This is one of the earliest papers that discussed pain and discomfort related to adjunctive orthodontic procedures. The oldest paper among the top 10 papers is written by Ngan et al. entitled “Perception of Discomfort by Patients Undergoing Orthodontic Treatment” [23]. Published in 1989, the article exclusively investigated patient‐reported discomfort during treatment, reflecting an early recognition of the value of patient experience in orthodontics. Collectively, the top‐cited articles laid the foundation for ORDP research, establishing core concepts and shaping the trajectory of subsequent investigations.

**Figure 6:**
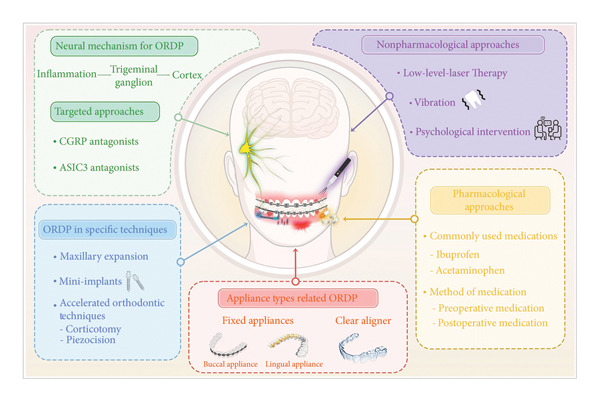
Integrated visualization of keyword clusters and trending topics in ORDP research. The five main sections are colored according to clustering analysis, while the contents represent key trends for discussion.

Particularly, we conducted a systematic keyword analysis of pertinent publications to explore the research hotspots and progress within the ORDP domain. On the basis of colored keyword clustering (Figure 4), time‐zone visualization (Figure 5(a)), and burst analysis (Figure 5(b)), we systematically categorized thematic developments into five critical areas of discussion and created a schematic illustration (Figure 6), where each color block represents a thematic domain.

### 4.1. Influence of Orthodontic Appliance Type on ORDP

Orthodontic treatment involves the utilization of diverse appliances that significantly impact patient comfort and pain perception. Our findings indicate that common orthodontic appliances include FAs, mainly comprising buccal and lingual appliances, and CAs, with Invisalign being the primary representative [24]. In recent years, CAs have emerged as a research hotspot. Compared to conventional FAs, CAs offer improved aesthetics, reduced discomfort, greater ease of use, and enhanced hygiene [25] and generally receive high levels of patient satisfaction [26]. These factors, coupled with their clinical efficacy for tooth movement [27], have driven the trend toward increased research and clinical adoption of CAs.

Both CAs and FAs follow a similar pattern of pain progression, with an initial peak in pain within 24 h and a return to baseline after 7 days [28]. However, patients treated with CAs report lower pain levels during the initial month. This may be attributed to the ability of CAs to alleviate pain by promoting blood flow through the periodontal ligament and the option to remove them from the teeth, in contrast to the continuous force exerted by FAs [29, 30]. In the first month, CA patients predominantly experience acute, sensitive, and throbbing pain, whereas FA patients mainly report sensitive pain [31]. This advantage diminishes after the first month, with CA patients subsequently experiencing increased pain levels [32]. FA patients face increasing pain levels with new orthodontic stimuli in revisits, especially when changing to new archwire materials [33]. In addition, CA patients experience the most pain at rest, whereas FA patients at chewing [34]. The pain locations also differ, with CA patients feeling pain primarily at the anterior mandible and FA patients at the anterior maxilla [29].

In addition, CAs and fixed lingual appliances, both considered aesthetically superior orthodontic appliances, have been compared in several studies regarding ORDP. Patients with fixed lingual appliances experience pain levels that are overall similar to those with CAs but report more discomfort. This discomfort includes difficulties with pronunciation (especially consonants “s” and “z”), swallowing, mouth opening, sleeping, taste changes, and increased food residue under the appliances [35, 36]. Moreover, patients wearing lingual appliances experience a greater impact on their OHRQoL compared to those wearing Invisalign appliances, even after the first 3 months [37]. Research has indicated that fixed lingual appliances receive significantly fewer positive reviews compared to CAs (Invisalign) [38]. Given these findings, future studies should focus on identifying patient characteristics that influence and predict ORDP, thereby enabling the selection of appropriate orthodontic appliances to effectively minimize ORDP.

### 4.2. Neural Mechanisms and Targeted Approaches of ORDP

Driven by continuous technological progress, the mechanisms of ORDP have been increasingly unraveled and more deeply understood in recent years. This is evident from the prominent presence of related terms such as “trigeminal ganglion” and “receptor” in the keyword co‐occurrence networks (Figure 4), highlighting their frequent occurrence in recent research.

The biological events during ORDP are complex and multifactorial. Orthodontic pain is a type of peripheral inflammatory pain that is transmitted mainly by the trigeminal nerve [39]. Upon local inflammation, nociceptive stimuli are generated, and tissue stress is transduced by periodontal sensory endings and transmitted to the trigeminal ganglion (TG) [39]. These stimuli are then relayed to the somatosensory cortex, resulting in changes in brain activity and structure [9]. Studies in recent years have highlighted alterations in brain regions following orthodontic pain, including changes in brain topological properties [40], similar brain activation patterns with or without placebo [41], and shifts in functional connectivity between gray and white matter networks [42]. In addition, research showed congruent expressions of pain at the behavioral and neuronal network levels during orthodontic pain [43].

Advanced comprehension of orthodontic pain mechanisms has led to the development of targeted therapeutic approaches. Calcitonin gene–related peptide (CGRP) antagonists, such as olcegepant, have shown promise in treating pain with a favorable safety profile [44]. In addition, Acid‐sensing ion channel 3 (ASIC3) antagonists, such as APETx2, and NGF‐neutralizing antibodies have shown potential in reducing tooth mechanical hyperalgesia induced by orthodontic tooth movement. This effect is achieved by modulating ASIC3 expression in the TG [45]. There is a need for greater interdisciplinary collaboration between dentistry and neuroscience to fully understand the neurological responses to orthodontic pain and to identify more effective management strategies.

### 4.3. ORDP in Specific Orthodontic Techniques and Accelerated Orthodontics

Orthodontic techniques such as maxillary expansion and mini‐implants are common in clinical practice. Maxillary expansion, which involves opening the midpalatal suture, is a well‐established technique for addressing maxillary constriction [46, 47]. The rate of expansion affects pain severity, with slow maxillary expansion resulting in lower pain levels than rapid maxillary expansion [48]. Rapid maxillary expansion is associated with frequent pain, particularly in the first week postplacement, with patients also experiencing discomfort, including speech, chewing, swallowing difficulties, and mucosal injuries [49]. Variations in appliance type may influence ORDP during maxillary expansion; for instance, the Leaf Expander has been shown to induce less pain than the Hyrax Expander in the initial week of treatment [50]. However, the current body of research presents conflicting outcomes regarding pain caused by different appliances in maxillary expansion [51]. Thus, more definitive studies are needed to identify the most comfortable and effective maxillary expansion appliances.

Mini‐implants are highly effective in orthodontic treatment for anchorage, preventing undesired tooth movement and associated complications [52]. Compared to traditional implants, mini‐implants tend to result in less pain [53]. Pain is noted to be most intense within the first 24 h postinsertion, thereafter diminishing and stabilizing after approximately 1 week [54]. Variability in the incidence of pain among patients has been observed, which may be attributed to differences in implant placement sites [55]. Discomfort is associated primarily with tissue swelling and the physical impact of the mini‐implants on the cheeks, especially when implanted in the posterior maxilla [52, 56]. Notably, many studies on patient‐reported outcome measures for temporary anchorage devices, including mini‐implants, have been found to have high risks of bias [56]. This highlights the urgent need for rigorous, standardized research to clarify the influencing factors of ORDP associated with mini‐implants.

Accelerated orthodontic techniques (AOTs) have garnered significant interest for their potential to reduce treatment duration while maintaining satisfactory outcomes [57]. However, surgical AOTs, including corticotomy and piezocision, are accompanied by different levels of pain and discomfort. Patient‐reported pain, typically described as mild to moderate, occurs 24 h postintervention and subsides to a lower level after a few weeks. Even 4 weeks after surgery, pain levels remain elevated compared to conventional orthodontic procedures [58]. Mucosal swelling is a common postoperative discomfort, which is likely a consequence of surgical incisions [59]. The ORDP of AOTs varies with the specific technique employed. A randomized controlled trial reported that patients who underwent flapless corticotomy, such as piezocision, experienced less pain and discomfort in the initial postoperative week. This is because flapless corticotomy causes less oral tissue trauma than conventional corticotomies, and the difference became statistically nonsignificant after 2–4 weeks [60]. A systematic review has indicated that the existing evidence on ORDP of AOTs is generally of low quality [61]. Thus, more high‐quality randomized controlled clinical trials are needed to validate the ORDP of AOTs, thereby guiding the selection of personalized AOT strategies to minimize ORDP in future clinical practice.

### 4.4. Orthodontic Pain Management by Pharmacological Approaches

The preoperative administration of analgesics, particularly ibuprofen and acetaminophen, is a widely adopted practice to preemptively reduce postprocedure pain severity [62]. This strategy is particularly beneficial for pediatric and adolescent patients, who often experience heightened dental anxiety [63]. Another pain management approach involves prescribing analgesics after bracket bonding and initial wire placement, aligning with the anticipated peak in pain. This approach is justified by the typical onset of ORDP within a few hours (typically 2–4 h) after force application, peaking at approximately 24 h [64]. However, studies comparing the two approaches are still lacking. Given the variability in ORDP among individuals, which is influenced by factors such as pain thresholds and the specific appliances used [65], more research on individualized selection and prescription of pharmacological pain relief is needed. Determining the optimal duration, dosage, frequency, and approach (pre‐, post‐, or combination) of analgesic administration based on individual patient clinical needs and regularly evaluating the efficacy of such administration to ensure optimal pain control will be beneficial to enhance patient experience.

### 4.5. Orthodontic Pain Management by Nonpharmacological Approaches

The use of NSAIDs and acetaminophen is often associated with adverse effects, including gastrointestinal complications, allergic reactions, elevated blood pressure, and hepatotoxicity [66, 67]. In light of these potential side effects, there is increasing interest in nonpharmacological strategies for pain management in orthodontics. Alternatives such as low‐level laser therapy (LLLT), mechanical vibration, masticatory adjuncts, and psychological interventions have demonstrated efficacy in alleviating orthodontic pain [68]. For example, the application of chewing gum or bite wafers, categorized as vibratory methods, has been shown to effectively reduce pain following archwire placement, particularly during the critical 2‐3 day period posttreatment [12]. These methods are thought to increase blood flow to the periodontal ligament, thereby preventing the accumulation of inflammatory metabolites that contribute to pain perception [69].

LLLT has attracted considerable attention, as demonstrated by the temporal trends and significant citation bursts in recent years (Figure 5). This surge is likely attributable to its noninvasive nature and economic advantages in orthodontic pain management. This therapy has been shown to reduce both the severity and frequency of painful symptoms without significant side effects. LLLT can rapidly alleviate pain associated with tooth separation, typically within 6–24 h [70–72]. Studies have confirmed that LLLT leads to a significant reduction in pain levels within 24–48 h following orthodontic procedures, a critical period characterized by heightened patient discomfort [73–77]. LLLT has also demonstrated effectiveness comparable to that of pharmacological treatments in terms of pain reduction in split‐mouth studies [78]. The underlying mechanism of LLLT involves retrograde mitochondrial signaling [79], which enhances cellular activity in both osseous and soft tissues, promotes reductions in pain episodes, and accelerates treatment duration [80].

The effectiveness of laser therapy in orthodontics is influenced by various parameters, including wavelength, output power, exposure time, energy density, and intensity. Most studies have employed LLLT with wavelengths ranging from 940 to 980 nm, which are known for their potential to penetrate deep into soft tissues, reaching the alveolar bone and periodontal ligament [81–84]. The diverse settings and protocols of irradiation techniques and laser parameters for LLLT in orthodontic pain management present an opportunity for further research [85]. By exploring these variations, the field can work toward establishing a standardized approach that maximizes the promising applications of laser therapy in clinical orthodontic practice.

These findings highlight the emergence of innovative and promising nonpharmacological alternatives for orthodontic pain management. Future clinical trials should be designed to explore the optimal protocols for these interventions and to investigate their potential synergies with conventional pharmacological approaches, thereby maximizing pain‐relieving effects and minimizing possible side effects.

### 4.6. Limitations

Given the strong positive correlations between citations from different databases (Web of Science, Scopus, Google Scholar, and PubMed) [86–88], our study focused exclusively on research from the Web of Science Core Collection to ensure consistency and readability of data statistics. However, this approach may have excluded some relevant publications available in other databases. The results could potentially vary if other databases were incorporated [89]. This could be an avenue for further research to complement this study. Another limitation is that self‐citations were not excluded in this study, which may result in some inflation of citation counts, particularly at the author level [90]. Current bibliometric software does not provide a reliable function for systematically filtering self‐citations, and given the size of our dataset (*n* = 970), manual identification was challenging. Future studies could address this limitation as more advanced tools and algorithms for self‐citation screening become available, or by performing sensitivity analyses when data volume permits.

## 5. Conclusions

This study reveals the global research distribution in the field of ORDP over time and across regions, highlighting key contributors across various dimensions. The field of ORDP has experienced significant growth, with emerging interests in invisible orthodontics, laser therapy for ORDP management, and AOTs. Given the current low level of evidence, further high‐quality research is needed to explore personalized appliance selection, optimize innovative orthodontic technique processes, and establish specific protocols for promising management strategies such as LLLT. Such research is anticipated to refine treatment protocols and improve patient experiences.

## Conflicts of Interest

The authors declare no conflicts of interest.

## Funding

This work was supported by the National Natural Science Foundation of China (62306193), the Sichuan Science and Technology Program (2024YFHZ0144 and 2023NSFSC0562), and the Network and Data Security Key Laboratory of Sichuan Province (NDS2024‐2).

## Supporting Information

Additional supporting information can be found online in the Supporting Information section.

## Supporting information


**Supporting Information 1** 1. Keyword analysis was refined by consolidating synonymous terms into a single representative term to ensure the accuracy of the analysis, with details provided in the Supporting Information (Table S1).

**Supporting Information 2** 2. The detailed keyword composition of each cluster is provided in the additional information (Table S2).

**Supporting Information 3** 3. Density map of the collaboration groups among institutions (Figure S1).

## Data Availability

The data that support the findings of this study are available in this article or its supporting information.
