# Supplementary material for: Research Hotspots and Emerging Trends of Orthodontic‐Related Discomfort and Pain: A Bibliometric Review
Source: Pain Res Manag. 2025 Dec 5;2025:3757286. doi: 10.1155/prm/3757286 (PMC12767382; doi:10.1155/prm/3757286)
Supplement: Supplementary file 1 — Supporting Information 1 1. Keyword analysis was refined by consolidating synonymous terms into a single representative term to ensure the accuracy of the analysis, with details provided in the Supporting Information (Table S1). [file PRM-2025-3757286-s002.docx]

| **Label** | **Replace by** |
| --- | --- |
| accelerated orthodontics | accelerated orthodontics |
| accelerated tooth movement | accelerated orthodontics |
| adolescent | adolescent |
| adolescents | adolescent |
| alignment | alignment |
| appliance | appliance |
| appliance therapy | appliance |
| appliances | appliance |
| archwire | archwire |
| archwires | archwire |
| bracket | brackets |
| brackets | brackets |
| c-fos | c-fos |
| c-fos expression | c-fos |
| clinical trial | clinical trail |
| clinical-trial | clinical trail |
| diode laser | diode laser |
| diode-laser | diode laser |
| elastomeric separation | elastomeric separation |
| elastomeric separators | elastomeric separation |
| experience | experience |
| experiences | experience |
| fixed appliance | fixed appliances |
| fixed appliances | fixed appliances |
| fixed orthodontic appliances | fixed appliances |
| fixed orthodontic treatment | fixed appliances |
| force | forces |
| forces | forces |
| implants | implants |
| dental implants | implants |
| laser | laser therapy |
| laser therapy | laser therapy |
| lllt | low-level laser therapy |
| low level laser therapy | low-level laser therapy |
| low-level laser | low-level laser therapy |
| low-level laser therapy | low-level laser therapy |
| low-level light therapy | low-level laser therapy |
| meta-analysis | meta-analysis |
| metaanalysis | meta-analysis |
| micro-osteoperforation | micro-osteoperforation |
| micro-osteoperforations | micro-osteoperforation |
| nonsteroidal antiinflammatory drugs | nsaids |
| nsaid | nsaids |
| nsaids | nsaids |
| oral health | oral-health |
| oral-health | oral-health |
| orthodontic appliance | orthodontic appliance |
| orthodontic appliances | orthodontic appliance |
| pain | pain |
| pain experience | pain |
| palatal expansion | palatal expansion technique |
| palatal expansion technique | palatal expansion technique |
| perception | perception |
| perceptions | perception |
| periodontal ligament | periodontal-ligament |
| periodontal-ligament | periodontal-ligament |
| photobiomodulation | photobiomodulation therapy |
| photobiomodulation therapy | photobiomodulation therapy |
| piezocision | piezocision |
| piezosurgery | piezocision |
| quality of life | quality-of-life |
| quality-of-life | quality-of-life |
| orthodontic | orthodontics |
| orthodontics | orthodontics |
| rat | rat |
| rats | rats |
| scale | scale |
| scales | scale |
| temporomandibular disorders | temporomandibular joint disorders |
| temporomandibular joint disorders | temporomandibular joint disorders |
| tmd | temporomandibular joint disorders |
| tooth movement | orthodontic tooth movement |
| orthodontic tooth movement | orthodontic tooth movement |
| trigeminal ganglia | trigeminal ganglion |
| trigeminal ganglion | trigeminal ganglion |
