# Supplementary material for: Research Hotspots and Emerging Trends of Orthodontic‐Related Discomfort and Pain: A Bibliometric Review
Source: Pain Res Manag. 2025 Dec 5;2025:3757286. doi: 10.1155/prm/3757286 (PMC12767382; doi:10.1155/prm/3757286)
Supplement: Supplementary file 2 — Supporting Information 2 2. The detailed keyword composition of each cluster is provided in the additional information (Table S2). [file PRM-2025-3757286-s003.docx]

| **Cluster 1** | **Cluster 2** | **Cluster 3** | | **Cluster 4** | **Cluster 5** |
| --- | --- | --- | --- | --- | --- |
| adolescent | activation | accelerated orthodontics | | apical root resorption | acetaminophen |
| anxiety | alveolar bone | adults | | blind | alignment |
| appliance | c-fos | age | | diode laser | analgesics |
| brackets | cells | anchorage | | double-blind | archwire |
| children | crevicular fluid | beam computed-tomography | | duration | chewing gum |
| clear aligners | cytokines | bone | | debonding | clinical trail |
| covid-19 | experimental tooth movement | canine retraction | | efficacy | discomfort |
| efficiency | expression | corticision | | elastomeric separation | fixed appliances |
| experience | forces | corticotomy | | intensity | ibuprofen |
| follow-up | gene-related peptide | en-masse retraction | | irradiation | initial alignment |
| health | gingival crevicular fluid | implants | | laser therapy | initial archwire placement |
| impact | inflammation | micro-osteoperforation | | level laser therapy | level |
| invisalign | interleukin-1-beta | mini-implants | | low-level laser therapy | meta-analysis |
| malocclusion | mechanisms | movement | | management | perception |
| maxillary expansion | model | piezocision | | nm | placebo |
| occlusion | neurons | rapid maxillary expansion | | photobiomodulation therapy | preoperative ibuprofen |
| oral health-related quality of life | nsaids | root resorption | | placement | randomized clinical-trial |
| oral-health | orofacial pain | skeletal anchorage | | reducing pain | vibration |
| orthodontic appliance | orthodontic pain | speech | | relief |  |
| orthodontic brackets | orthodontic tooth movement | stability | | systematic review |  |
| orthodontic treatment | periodontal-ligament | stress | | therapy |  |
| orthodontics | prostaglandin-e | surgery | | velocity |  |
| orthognathic surgery | rat | teeth | |  |  |
| pain | receptor | traumatic ulcers | |  |  |
| pain perception | responses | visual analog scale | |  |  |
| palatal expansion technique | stimulation | |  |  |  |
| prevalence | substance-p | |  |  |  |
| quality-of-life | trigeminal ganglion | |  |  |  |
| questionnaire |  |  | |  |  |
| reliability |  |  | |  |  |
| satisfaction |  |  | |  |  |
| scale |  |  | |  |  |
| self-esteem |  |  | |  |  |
| symptoms |  |  | |  |  |
| temporomandibular joint disorders |  |  | |  |  |
| trial |  |  | |  |  |
| validation |  |  | |  |  |
| validity |  |  | |  |  |
