# Supplementary figures and images for: Research Hotspots and Emerging Trends of Orthodontic‐Related Discomfort and Pain: A Bibliometric Review
Source: Pain Res Manag. 2025 Dec 5;2025:3757286. doi: 10.1155/prm/3757286 (PMC12767382; doi:10.1155/prm/3757286)

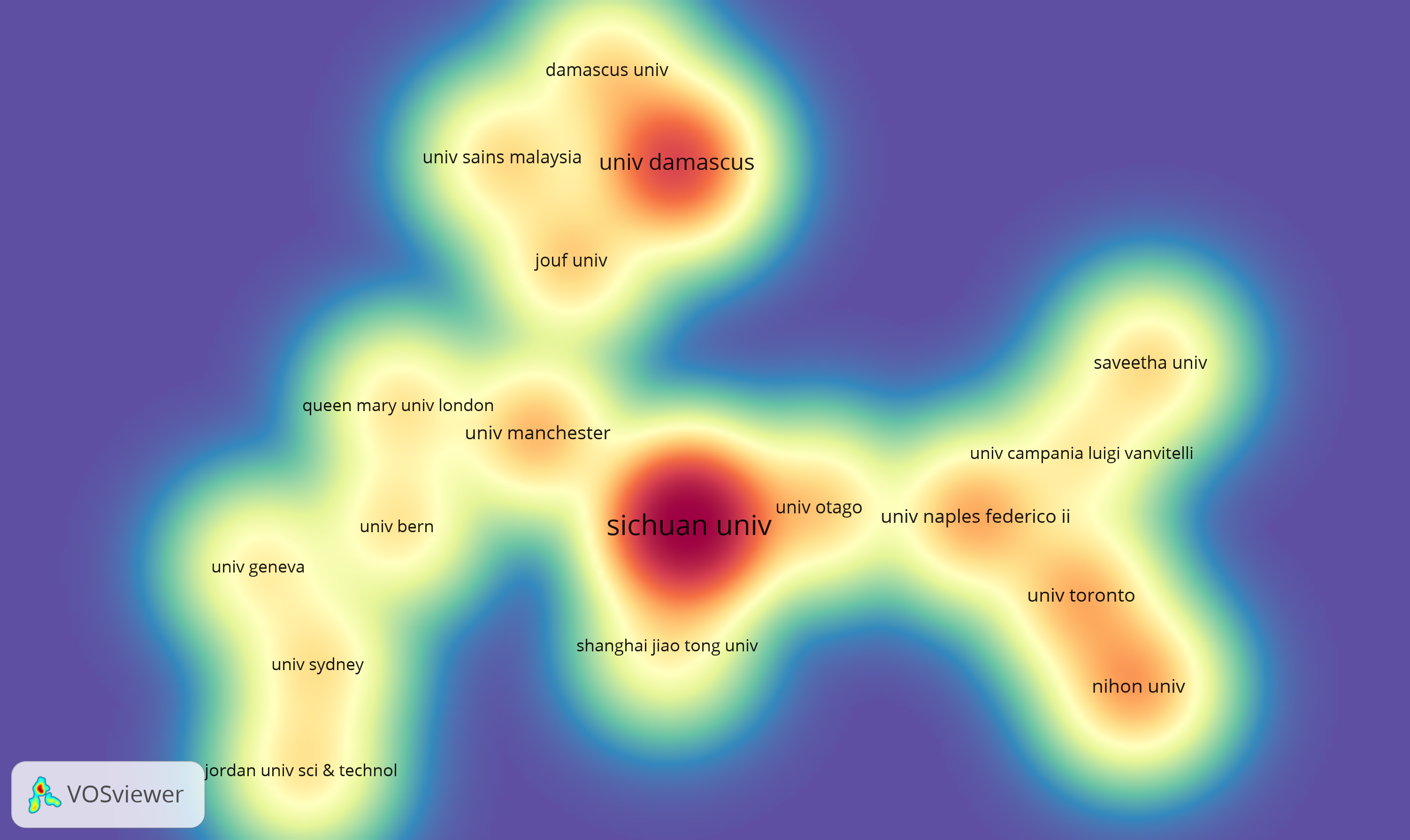

Supplement: Supplementary file 3 — Supporting Information 3 3. Density map of the collaboration groups among institutions (Figure S1). [file PRM-2025-3757286-s001.tif]
